# Role of *Notch* gene receptors as prognostic biomarkers in colorectal cancer

**DOI:** 10.1038/s41598-025-00424-5

**Published:** 2025-09-30

**Authors:** Abhay Kumar Sharma, Nimisha Nimisha, Arun Kumar, Apurva Apurva, Abhishek Kumar, Ejaj Ahmad, Asgar Ali, Birendra Prasad, Sundeep Singh Saluja

**Affiliations:** 1 Department of Botany/Biotechnology, Patna University Patna, Patna, India; 2 Central Molecular Lab, Govind Ballabh Pant Institute of Postgraduate Medical Education and Research (GIPMER), New Delhi, India; 3 Department of Biotechnology, Amity University Noida, Noida, India; 4 Department of Biochemistry, All India Institute of Medical Sciences Patna, Patna, India; 5 Department of G I Surgery, Govind Ballabh Pant Institute of Postgraduate Medical Education and Research (GIPMER), 1, Jawaharlal Nehru Marg, 64 Khamba, Raj Ghat, New Delhi, India

**Keywords:** Colorectal cancer, Single nucleotide polymorphism, *Notch*, PCR–RFLP, Western bloting, Survival, Cancer, Molecular biology

## Abstract

The incidence of colorectal cancer (CRC) is increasing across the world, especially in younger age groups and the Indian subcontinent. Dysregulation in Notch pathway genes and their Single Nucleotide Polymorphism (SNPs) can potentially lead to aberrant signaling and contribute to CRC development. We investigated SNPs of *Notch1* (rs3124591), *Notch2* (rs10910779), *Notch3* (rs1043994), and *Notch4* (rs367398) in CRC (n = 103) and Controls (n = 103) along with their protein expression in the cases. The SNPs were detected by Polymerase Chain Reaction–Restriction Fragment Length Polymorphism method followed by Sanger sequencing. The protein expression was determined by the western blot technique. SPSS was used to analyze the correlations of the molecular findings with clinicopathological features and survival. The frequency of CT genotype in *Notch1* was significantly lower in CRC patients compared to healthy controls (40.77% vs 62.14%; *p* = 0.009) and was associated with increased depth of invasion (*p* = 0.03). *Notch3* polymorphism A > G showed significant association with the advanced TNM stage (*p* = 0.013). Interestingly, AG and GG genotype in *Notch3* was significantly associated with increased protein expression (*p* = 0.047). The patients carrying the 'G' allele in *Notch3* had an increased risk of having CRC (OR = 1.697, CI 95%: 1.001–2.873, *p* = 0.049) and a lower survival rate (*p* > 0.05). *Notch4* polymorphism showed an association with tumor grade (*p* = 0.03). The genotypes CT and TT had lower survival rates than the CC genotype (*p* = 0.08). *Notch* receptor polymorphism, especially *Notch3,* is associated with increased protein expression and a higher risk of having CRC. Furthermore, poor survival in patients with *Notch3* and *Notch4* polymorphism suggests their potential as prognostic biomarker in CRC.

## Introduction

Colorectal cancer (CRC) is among the most common gastrointestinal malignancy, with a high rate of cancer-related death globally. The incidence of CRC was estimated to be 9.6% of all new cases diagnosed while 9.3% of all deaths related to cancer reported worldwide; hence, it has ranked 3^rd^ most common cancer and 2^nd^ most common cause of cancer-related mortality^1^. The incidence of CRC is increasing in developing countries that were once considered low prevalence. In India, CRC-related morbidity and mortality was reported to be 5% and 4.5% respectively^2^.

Epidemiological studies suggest that alcohol intake, smoking, high consumption of red and processed meat, and genetic variations are associated with an increased risk of developing CRC^3,4^. Genome-wide association studies (GWAS) suggest the role of genetic variants in increased risk for developing CRC, affecting its pathogenesis and survival^5,6^. Single nucleotide polymorphisms (SNPs) are the most prevalent genetic variations, which occur in every 100 to 300 bases and account for around 90% of all genetic variation in humans^7^. It may be found in different locations of a gene, such as exon, intron, and promoter region, as well as in their 5′ and 3′ UTRs. The changes in gene expression depend on the location of the SNPs in the gene^8,9^. When these changes cause alteration in signal transduction pathways, create genomic instability or result in loss of cell cycle control, tumor formation may be promoted.

The Notch signalling pathway is an important signal transduction pathway involving cancer stem cells^10^. It has a role in various cellular processes such as epithelial cell polarity/adhesion, apoptosis, and regulation of cancer cells by maintaining self-renewal and multidirectional differentiation^11,12^. It has four transmembrane Notch receptors (Notch1-4) that interact with five ligands (Jagged 1–2 and Delta-like 1,3,4)*.* Notch receptors are transmembrane proteins containing heterodimer domains expressed on the cell surface, while ligands are homodimer proteins of the Delta/Serrated/Lag2 (DSL) family that contain EGF-like repeats. Activation of this signalling occurs by binding Notch ligands to the EGF-like repeats receptors on the neighbouring cells^13^. Subsequently activation of the γ-secretase protein complex results in cleavage of the notch receptors into an active form of *Notch* intracellular domain (NICD)^14^. NCID is then translocated towards the nucleus, where it binds with other signalling proteins along with transcription factors, which consequently regulates *Notch*-targeted genes^15^.

The SNPs in key genes of the Notch pathway can affect the signalling of this pathway, resulting in the development of cancer. The genetic variations in Notch have been linked to the development and progression of different solid tumors, including breast cancer, liver, CRC, lung, and glioma^16–22^. The study of SNPs in the Notch pathway might help us to know a predictable risk for the development of CRC^19–22^. Therefore, the present study explored the association of SNPs of *Notch1* (rs3124591), *Notch2* (rs10910779), *Notch3* (rs1043994), and *Notch4* (rs367398) and CRC in the Indian population. We also evaluated the association between genetic alterations, clinicopathological characteristics and survival patterns.

## Material and methods

### Patient population and sample collection

A total of 103 patients with histo-pathologically confirmed CRC and an equal number of age and sex-matched healthy controls unrelated to cases were enrolled in the study. Patients who had received neoadjuvant treatment before the sample collection or those who had dual malignancies, as well as those with age < 18yrs were exclude. Tumor and normal tissue from CRC cases were obtained either at colonoscopy or at the time of surgery. Tissue samples were collected in a nuclease-free collection tube containing 1X phosphate buffer saline (pH-7.4) and stored at -80 °C. Whole blood (2 ml) was collected in Ethylene diamine tetra acetic acid (EDTA) containing vacutainers from CRC patients and healthy volunteers. Informed consent was taken from all participants. Clinical and pathological details were recorded. The clinical tumor stage was classified according to the American Journal of Cancer Classification (AJCC).

### DNA extraction

Genomic DNA was extracted from tumor and normal tissue collected from CRC patients and whole blood of healthy control using the Wizard Genomic DNA purification kit (Promega; A1125) as per manufacturer’s instruction, with minor modifications. The purity of isolated DNA was checked by taking absorbance at 260/280 nm on NanoDrop one spectrophotometer (Thermo Fischer, New York), and quantification was done by Qubit 4 fluorometer (Invitrogen, USA) using dsDNA BR Assay Kits (Invitrogen; Q32850). Further, the integrity of DNA was checked by agarose gel electrophoresis (0.8%), and the bands were visualized under Chemi-Doc (Bio-Rad).

### Whole exome sequencing

Whole-exome libraries were prepared using the Twist EF 2.0 kit following the standard Twist target enrichment protocol (Twist Bioscience, South San Francisco, CA). DNA fragmentation, end-repaired, and dA-tailed was achieved using the enzymes and buffers provided in the kit. Paired-end adaptors were ligated, and libraries were purified and amplified with UDI primers. Further genomic libraries were pooled and hybridized with Twist oligo probe at 70 °C for 15–16 h. Hybridized targets were captured using streptavidin beads and amplified using PCR. Finally, libraries were purified and quantified with 1 × dsDNA HS Qubit, and size-validated using an Agilent Bioanalyzer High Sensitivity DNA kit. Sequencing was performed on an Illumina NextSeq 2000 platform with 2 × 151 bp reads. FASTQ files were generated using Illumina DRAGEN BCL Convert 4.2.7.

To identify common SNPs from our in-house sequencing data (n = 14), raw sequencing reads were quality-checked using FastQC. Trimmomatic was used for adapter trimming and low-quality read filtering. The cleaned reads were then aligned to the human reference genome (GRCh38) using BWA-MEM, and duplicate reads were marked. Variant calling was conducted using GATK HaplotypeCaller, generating a VCF file of potential SNPs. The resulting variants are filtered based on quality metrics and annotated using dbSNP.

### Polymerase chain reaction-restriction fragment length polymorphism (PCR–RFLP)

In the present study, Notch receptor genes (*Notch 1–4*) were assessed to determine the effects of their variants on the risk of CRC. Genotyping of the *Notch* gene was assessed using restriction fragment length polymorphism. The restriction site of *Notch1, Notch2, Notch3,* and *Notch4* was selected from previous studies and the NCBI SNP search tool (https://www.ncbi.nlm.nih.gov/snp/?term=). The primer sequence of each SNP was designed using the NCBI Primer blast tool for *Notch1* (F:5'-TAACAGGCAGGTGATGCTGG-3', R: 5' CCGACC AGAGGAGCCTTTTT-3'), *Notch2* (F: 5'-ACATCGAGACCCCTGTGAGA-3', R: 5'-AGCTGGGGGACATTTAAGAGC-3'), *Notch3* (F: 5'-TAGTCGGGGGTGTGGTCAGT-3', R: 5'-CCTCTGACTCTCCTGAGTAG-3'), *Notch4* (F: 5'-TAGTGTTCCTCCACTCTTCC TC-3', R: 5'AGTGAAGGGGGCTGCATTCCAC-3'). PCR was carried out in a 20 µl reaction volume containing 1X PCR buffer,1.5 mM MgCl_2_, 200 µM dNTPs, 0.3 µM forward and reverse primers,100 ng genomic DNA, and 0.5U *Taq* DNA polymerase. The PCR amplicon was amplified in a thermal cycler (Bio-Rad, USA); initial denaturation at 95 °C for 7 min, followed by 35 cycles; denaturation at 95 °C for 30 s, annealing at 57 °C for 35 s and extension at 72 °C for 30 s followed by the final extension at 72 °C for 7 min. The size of the PCR product was checked on 2% agarose gel and visualized under ChemiDoc. Amplified product was subsequently digested by the restriction enzyme (New England Biolab). Restriction digestion was carried out in a 20 µl reaction volume containing 10 µl amplified PCR product, 1X restriction digestion buffer, and 2.5U restriction enzyme. The reaction mixture was incubated at 37˚C for 1 h, followed by heat inactivation at 65˚C for 15 min. Finally, the digested PCR product was assessed on 3% agarose gel and visualized under UV ChemiDoc. Restriction enzyme used in genotyping and their digested product size are summarized in supplementary table 1. The PCR–RFLP results were further validated by Sanger Sequencing. The genotype pattern of *Notch1, 2,* and *3* genes were further confirmed by the automated Sanger sequencing (Applied Biosystems, Inc., Foster City, CA, USA) from Lifetech Service Laboratory Invitrogen Bioservices India Pvt. Ltd.

### Sanger sequencing

The PCR amplicons of *Notch1*, *Notch3,* and *Notch4* were sequenced with the same primer used in PCR–RFLP analysis to avoid errors in genotyping. The sequenced data was analyzed using Finch TV chromatogram viewer followed by the pairwise alignment of nucleotide with GRCh38. 14 primary assembly database sequence available on National Center for Biotechnology Information (NCBI) for nucleotide variations (https://blast.ncbi.nlm.nih.gov/Blast.cgi?PROGRAM=lastnandPAGE_TYPE=lastSearchandBLAST_SPEC=ndLINK_LOC=blasttabandLAST_PAGE=blastx) and https://blast.ncbi.nlm.nih.gov/Blast.cgi?PROGRAM=blastxandPAGE_TYPE=BlastSearchandLINK_LOC=blasthome.

### Western blot

Protein was extracted using tissue protein extraction reagent (T-PER, Thermo Scientific, cat-78510) following the manufacturer’s instructions. The concentration of protein was measured using a Pierce BCA protein assay kit (Thermo Scientific; 23,225). The estimated protein of 25 µg was separated on 12% sodium dodecyl sulfate–polyacrylamide gel electrophoresis (SDS-PAGE) and then electrophoretically transferred on nitrocellulose membranes. The membranes were saturated in 5%blocking buffer (non-fat dried milk) for 1 h at room temperature, membranes were washed with 1X TBST and then membrane were incubated with primary antibody of Notch1 (CST; #3608) Notch2 (CST; #5732) Notch3 (Abcam; ab23426) and Notch4 (Abcam; ab184742) overnight at 4°Ct. The β-actin (Affinity; #AF7018) was used as an internal control. After incubation, membranes were washed with 1X TBST, followed by incubation with HRP-linked secondary antibody (CST; #7074) for 1 h at room temperature. Again, after washing with 1X TBST, the protein bands were detected using Pierce ECL Plus reagent (Thermo Fisher Scientific, USA) and visualized under Chemi doc (Bio-Rad Laboratories Inc.).

### Survival analysis

The follow-up of patients was done every three months in 1st year and then every six months till the date of survival analysis, either through telephonic conversation or a hospital OPD visit. The Kaplan–Meier log-rank test was used to calculate the mean survival time and assess the effect of Notch polymorphism on survival.

### Statistical analysis

The genotype and allele frequency for all SNPs and test for deviation from Hardy Weinberg Equilibrium (HWE) were performed by using the online available tool https://www.had2know.org/academics/hardy-weinberg-equilibrium-calculator-2-alleles.html and https://www.snpstats.net/ The allelic association the protein expression was analyzed using ‘epitools’ and ‘pwr’ R packages. The other Statistical analysis for association with clinicopathological parameters was accomplished using IBM Statistical Package for Social Sciences (SPSS) statistics 24.0 version software. The genetic association of each SNPs with CRC patients were evaluated by case–control comparison using the Pearson chi-square (χ^2^) test, odds ratio (OR), and 95% confidence interval (CI). A *p*-value ≤ 0.05 (2-sided) was considered significant for all the cases.

## Results

### Demographical and clinical parameters

One hundred three CRC patients and an equal number of age-sex-matched healthy controls were taken for RFLP. The mean age of patients was 50.59 ± 13.61 years compared to the healthy control, 49.8 ± 12.71 years, respectively. Approximately 25% of patients were less than 40 years old, with a similar male–female distribution. Among the 103 cases, 59 were male and 44 were female. In the control group55were, male and 48 were female. The clinicopathological characteristics of CRC patients are summarised in supplementary table 2.

### Notch Genotyping: *Notch3* as a candidate gene for CRC risk at rs1043994

**Fig. 1:**
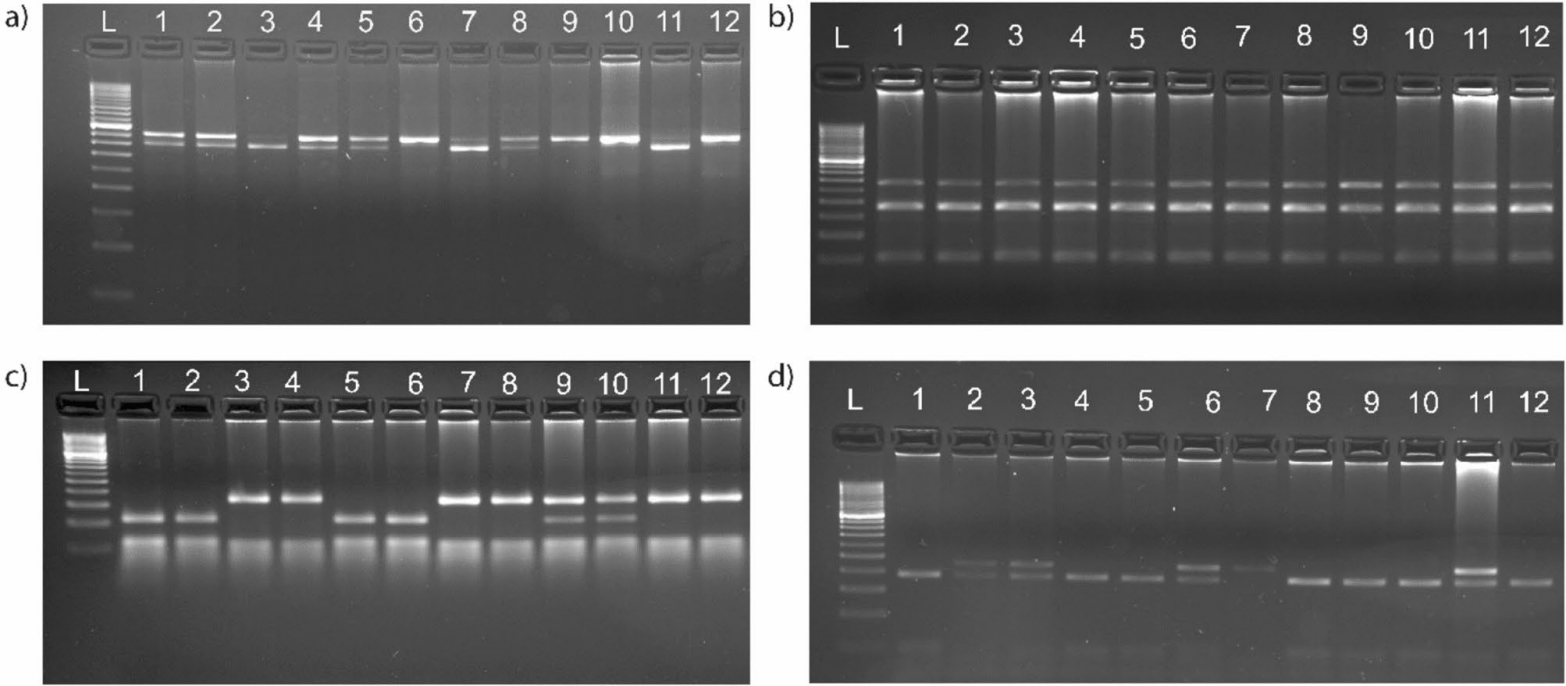
Representative image of PCR–RFLP: (**a**) *Notch1*; lane 3 and 11: CC genotype (397 and 43 bp) lane 6, 9, 10, and 12 TT genotype (440 bp) and lane 1, 2, 4, 5, and 6: TC genotype (440, 397, and 43 bp) (**b**) *Notch2*; lane 1–12 showing TT genotype (228 and 100 bp) (**c**) *Notch3*; lane 1,2,5, and 6 GG genotype (107 and 61 bp), lane 3,4,7,8,11, and 12 AA genotype (168 and 61 bp), lane 9 and 10: GA genotype (168 bp, 107 bp, and 61 bp) (**d**) *Notch4*; lane 1,4,5,8,9,10, and 12: CC genotype (190 and 40 bp), lane 2,3, and 6 CT genotype (230, 190, and 40 bp) and lane 7: TT genotype (230 bp) in different samples. L-50 bp DNA ladder.

Genotyping was analyzed by PCR–RFLP method. The amplified PCR product and digested pattern of Notch1-4 are shown in Fig. 1. Here, we did not detect any difference between the tumor and normal tissue, suggesting that somatic change was noted in the Notch receptors. The distribution of genotype frequencies of *Notch1* and *Notch4* followed Hardy–Weinberg equilibrium (HWE), while the frequency of *Notch3* showed deviation from HWE (*p* = 0.046). In healthy controls, the frequency distribution of *Notch1* deviated from HWE, while *Notch3* and *Notch4* followed HWE (Supplementary Table 3). The *Notch2* genotype data showed no polymorphism. The genotype and allele frequencies of Notch1-4 polymorphism among the patients and control groups are shown in Table 1. The genotype frequency of CT type in *Notch1* was significantly lower in CRC patients compared to controls (40.77% vs 62.14; *p* = 0.0002), further, an OR = 2.11 (95% CI: 1.20–3.72) suggests an associated increased risk (Table 1). We also found differences in genotype frequency (AA, AG, and GG) between the CRC and healthy controls for *Notch3* polymorphism (67%, 25.2%, and 7.8% vs 77.7%, 18.4% and 3.9%). These results suggested homozygous (GG) and heterozygous (AG) genotypes were associated with increased risk of CRC. The G allele in *Notch3* was associated with an increased risk of developing CRC. The genotype distribution of *Notch4* gene polymorphism CC, CT, and TT was similar in cases and control (56.31%, 35.92%, 7.76%, 62.13%, 32.30%, and 5.82%).

**Table 1:** Genotype and allelic frequency distribution across different models of inheritance of *Notch1-4* in cases compared to controls.

Model	Genotype	Cases (n = 103)	Controls (n = 103)	OR (95% CI)	*p*
Notch 1 (rs3124591)					
Codominant	C/C	54 (52.4%)	39 (37.9%)	1.00	0.0002
	C/T	42 (40.8%)	64 (62.1%)	2.11 (1.20–3.72)	
	T/T	7 (6.8%)	0 (0%)	0.00 (0.00-NA)	
Dominant	C/C	54 (52.4%)	39 (37.9%)	1.00	0.035
	C/T-T/T	49 (47.6%)	64 (62.1%)	1.81 (1.04–3.15)	
Recessive	C/C–C/T	96 (93.2%)	103 (100%)	1.00	0.0016
	T/T	7 (6.8%)	0 (0%)	0.00 (0.00-NA)	
Overdominant	C/C-T/T	61 (59.2%)	39 (37.9%)	1.00	0.0021
	C/T	42 (40.8%)	64 (62.1%)	2.38 (1.36–4.17)	
Allele	CT	150 (72.82) 56 (27.58)	142 (68.93) 64 (31.07)	1.000.828 (0.541–1.268)	0.385
Log-additive	–	–	–	1.28 (0.79–2.10)	0.32
Notch 3 SNP (rs1043994)					
Codominant	A/A	69 (67%)	80 (77.7%)	1.00	0.2
	A/G	26 (25.2%)	19 (18.4%)	0.63 (0.32–1.24)	
	G/G	8 (7.8%)	4 (3.9%)	0.43 (0.12–1.49)	
Dominant	A/A	69 (67%)	80 (77.7%)	1.00	0.086
	A/G-G/G	34 (33%)	23 (22.3%)	0.58 (0.31–1.08)	
Recessive	A/A-A/G	95 (92.2%)	99 (96.1%)	1.00	0.23
	G/G	8 (7.8%)	4 (3.9%)	0.48 (0.14–1.65)	
Overdominant	A/A-G/G	77 (74.8%)	84 (81.5%)	1.00	0.24
	A/G	26 (25.2%)	19 (18.4%)	0.67 (0.34–1.31)	
Allele	AG	164 (79.62) 42 (20.38)	179 (86.90) 27 (13.10)	1.00 1.697 (1.001–2.873)	0.049
Log-additive	–	–	–	0.64 (0.40–1.05)	0.071
Notch 4 SNP (rs367398)					
Codominant	C/C	58 (56.3%)	64 (62.1%)	1.00	0.67
	C/T	37 (35.9%)	33 (32%)	0.81 (0.45–1.46)	
	T/T	8 (7.8%)	6 (5.8%)	0.68 (0.22–2.08)	
Dominant	C/C	58 (56.3%)	64 (62.1%)	1.00	0.39
	C/T-T/T	45 (43.7%)	39 (37.9%)	0.79 (0.45–1.37)	
Recessive	C/C–C/T	95 (92.2%)	97 (94.2%)	1.00	0.58
	T/T	8 (7.8%)	6 (5.8%)	0.73 (0.25–2.20)	
Overdominant	C/C-T/T	66 (64.1%)	70 (68%)	1.00	0.56
	C/T	37 (35.9%)	33 (32%)	0.84 (0.47–1.50)	
Allele	CT	153 (74.27) 53 (25.72)	161 (78.15) 45 (21.84)	1.00 1.043 (0.647–1.681)	0.862
Log-additive	–	–	–	0.82 (0.52–1.27)	0.37

### Association between *Notch* SNPs and clinico-pathological characteristics

We looked at the association between clinical characteristics, demographic features, and genotype distribution of *Notch1, 3,* and *4* genes in CRC patients to evaluate whether *Notch* polymorphism played any role in the disease progression. *Notch1* showed a significant association with tumor depth (*p* = 0.035). C > T transition in *Notch1* was associated with T3-T4 depth of invasion. The rs1043994 polymorphism in the *Notch3* gene showed a statistically significant association with TNM stage (*p* = 0.013), and 75% of the mutant genotype GG was found in stage III. This result indicates that genotype GG may be associated with a locally advanced disease stage. The SNP rs367398 in *Notch4* shows a significant association with the grade of differentiation (*p* = 0.039). CC type had a higher incidence of poorly differentiated tumors than other genotypes (Table 2).

**Table 2:** Clinico-pathological correlation with *Notch1, 3,* and* 4* Polymorphism in CRC patients.

Clinico-pathological parameters	*Notch1*				*Notch3*				*Notch4*			
	CC	CT	TT	*p*	AA	AG	GG	*p*	CC	CT	TT	*p*-value
Age (years)												
≤ 40	11	13	03	0.29	15	09	03	0.33	1642	1027	0107	0.65
> 40	43	29	04		54	17	05					
Gender												
Male	29	27	3	0.42	38	15	06	0.55	3424	2116	0404	0.89
Female	25	15	4		31	11	02					
Site												
Colon	37	261	03	0.38	47	15	04	0.44	3622	2512	0503	0.85
Rectum	17	6	04		22	11	04					
Grade of differentiation												
Well	05	07	00	0.66	07	02	03	0.15	07	02	03	0.03
Moderate	42	29	06		54	19	04		40	32	05	
Poor	07	06	01		08	05	01		11	03	00	
TNM stage												
I	01	07	02	0.12	08	00	02	0.01	09	01	00	0.10
II	24	18	02		34	10	00		26	15	03	
III	25	14	03		21	15	06		21	18	03	
IV	04	03	00		06	01	00		02	03	02	
Tumor Depth												
T1	00	03	00	0.03	02	00	01	0.19	03	00	00	0.19
T2	03	04	02		08	00	01		08	01	00	
T3	33	30	04		44	20	03		37	25	05	
T4	18	05	01		15	06	03		10	11	03	
Lymph node												
Positive	25	15	01	0.20	26	11	04	0.76	2137	17	03	0.63
Negative	29	27	06		43	15	04			20	05	
LNI												
Positive	12	10	01	0.85	18	04	01	0.42	14	07	02	0.82
Negative	42	32	06		51	22	07		44	30	06	
PNI												
Positive	07	06	00	0.57	09	03	01	0.98	10	02	01	0.23
Negative	44	36	07		60	23	07		48	35	07	

### Validation of RFLP of *Notch1, 3* and *4* by sanger sequencing

**Fig. 2:**
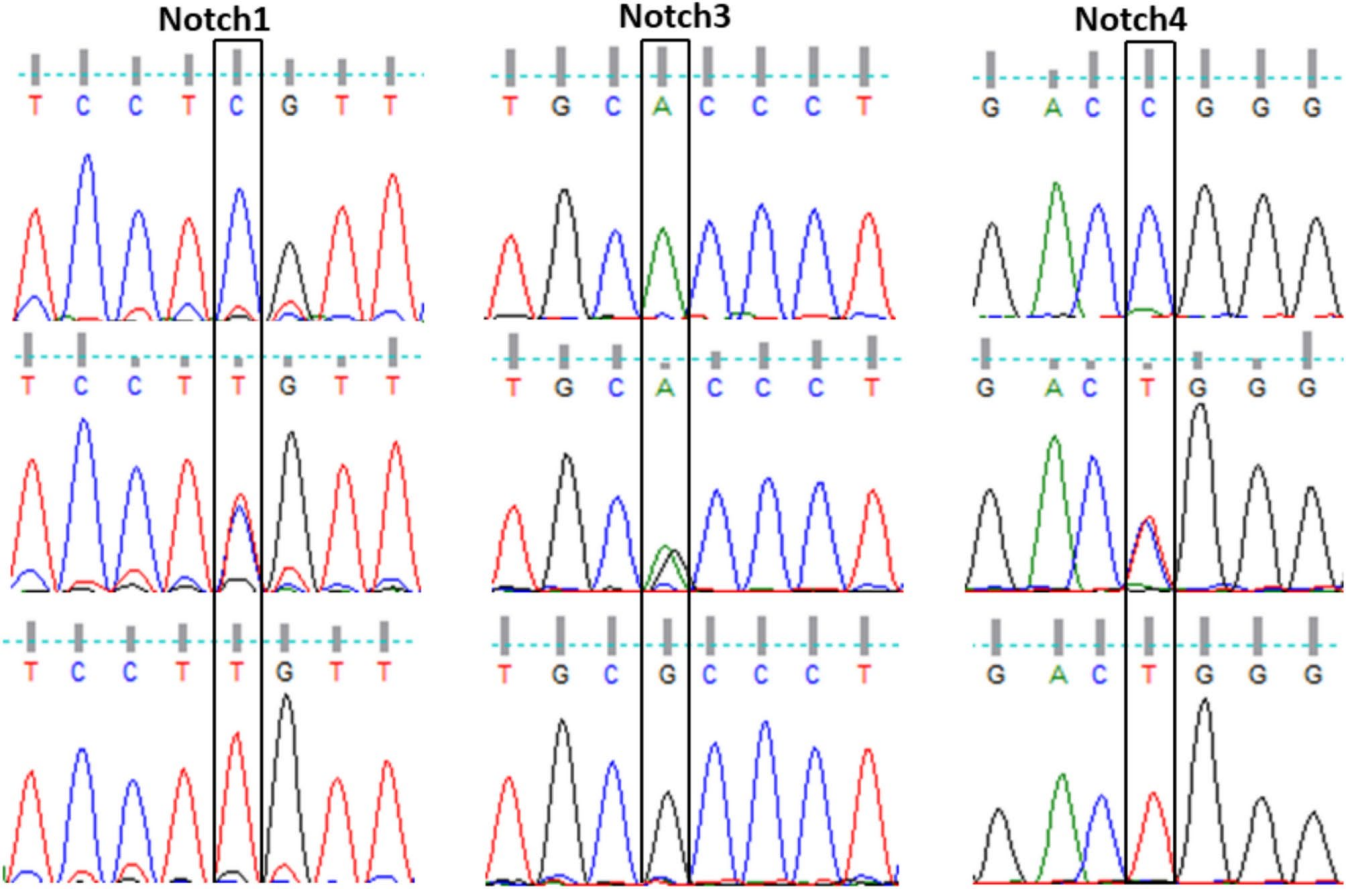
Electropherogram of Sanger sequencing obtained from the *Notch1*, *Notch3,* and *Notch4* PCR products. The chromatogram box indicates the changes in the nucleotide sequence at the polymorphic site.

The polymorphic change of *Notch* genes was confirmed by Sanger sequencing. The electropherogram representation of three different genotypes of *Notch1*, *Notch3,* and *Notch4* are shown in Fig. 2. Since the polymorphic sites, rs3124591 is located at 3’ UTR (*Notch1*), rs1043994 is a synonymous variant (*Notch3*), and rs367398 is located at 5’ UTR (*Notch4*), no change in the amino acid sequence was observed.

### Outcome of whole exome sequencing

**Fig. 3:**
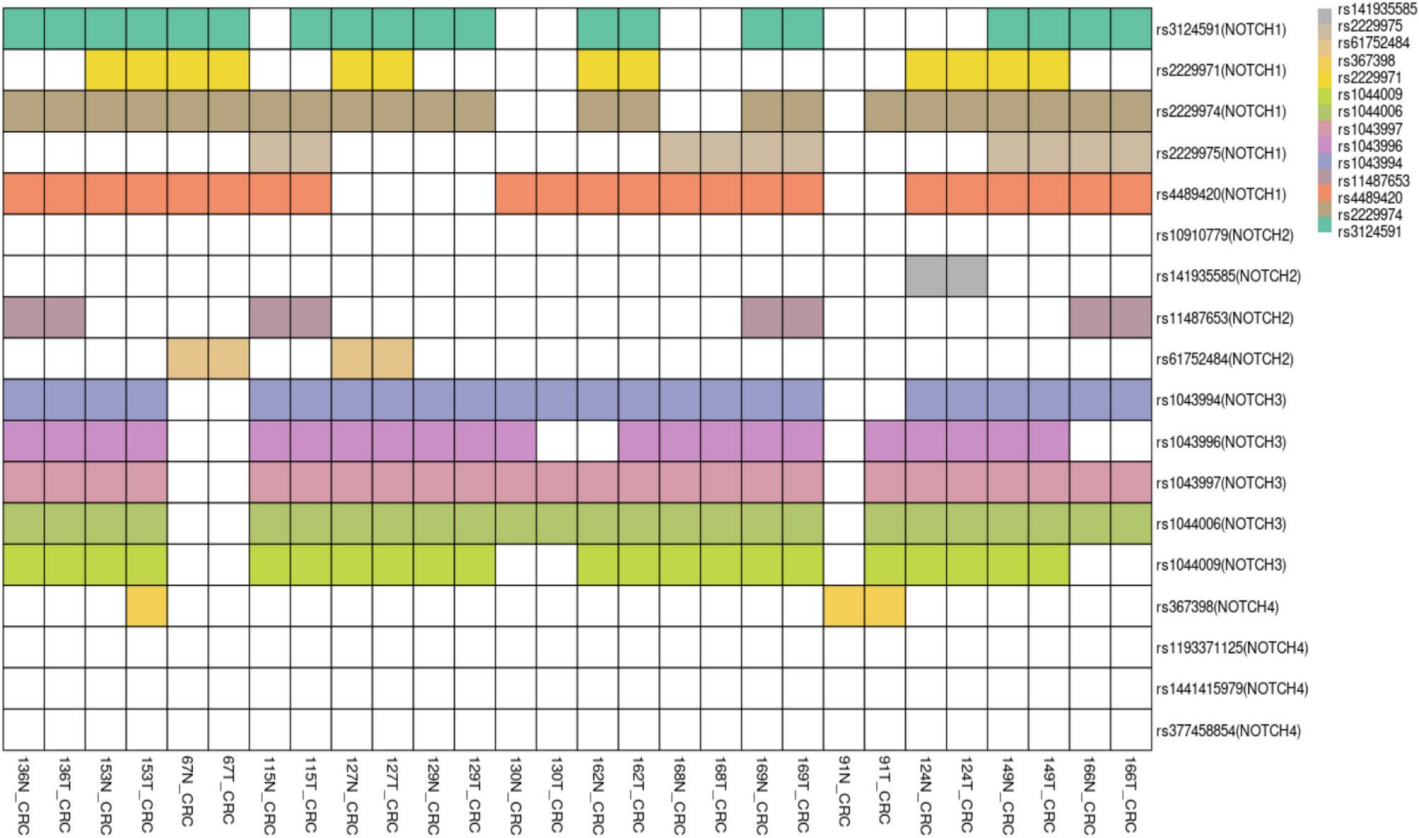
Heat Map representing common SNPs in the Notch family.

The common SNPs in the Notch family in colorectal cancer are shown in the Heat Map (Fig. 3). In Notch1, rs3124591, rs2229974, and rs4489420 were the most common SNPs noted in WES data. The findings supported our selection of Notch1 SNP (rs3124591), which has been commonly studied in solid tumors, including breast and colorectal cancer. Similarly, in Notch2, our sequencing results confirmed a monomorphic nature of SNP (rs10910779), supporting our similar findings from RFLP and Sanger sequencing. Although other Notch2 SNP were found in our population, they were comparatively less frequent, suggesting their weak association with CRC. In addition to rs1043994 in Notch 3, other SNPs (rs043996, rs1043997, rs1044006, and rs1044009) were commonly observed in our sequencing data. Similarly, in Notch4, the SNP (rs367398) that we selected for this study was the most common.

### Protein expression and its correlation with polymorphism

**Fig. 4:**
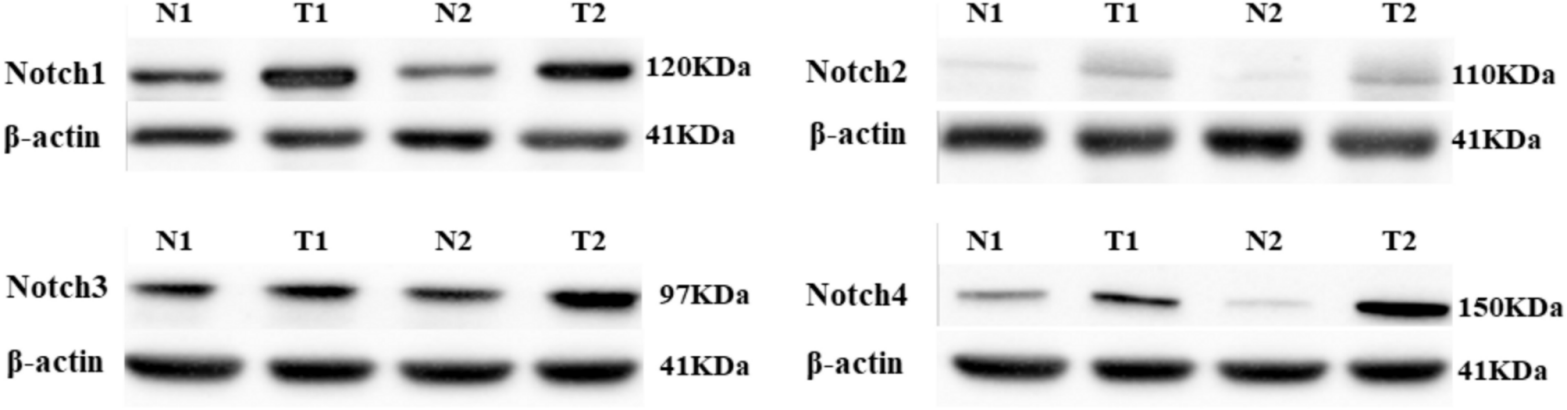
Protein expression Notch1-4 in normal and tumor CRC tissue analyzed by western blot technique. N and T indicate normal and tumor, respectively. Beta-actin was used as an internal control.

The protein expression of Notch1-4 in tumor and normal tissue was detected by western blot techniques (Fig. 4). We found that protein expression was upregulated in 43.57%, 45%, 56.31%, and 42.71% of tumor tissue in Notch1, Notch2, Notch3, and Notch4, respectively. We further correlated the protein expression with the genotype. In Notch1 and Notch4, we did not find a significant correlation with the mutant genotype. On the other hand, *Notch3* polymorphism rs1043994, A > G was significantly associated with the expression of Notch3 protein in tumor tissue and increased the risk of CRC development (Table 3). Interestingly, the AG and GG genotype had a higher expression level than the AA genotype. Meanwhile, the G allele of *Notch3* polymorphism was increased (~ threefold) in patients with upregulated Notch3 protein expression (OR = 2.62 (95% CI: 1.20–3.72, p = 0.01) (Table 3).

**Table 3:** Association between alleles of *Notch* polymorphisms and protein expression.

Gene	Allele	Up	Down	OR (95% CI)	*p*	Sensitivity	Specificity	Power
Notch1	C	71	79	1.00	1.00	0.724	0.269	0.36
	G	27	29	0.96 (0.52–1.78)				
Notch3	A	85	79	1.00	0.01	0.267	0.878	1.00
	G	31	11	2.62 (1.23–5.56)				
Notch4	C	64	89	1.00	0.74	0.273	0.754	0.99
	T	24	29	1.15 (0.61–2.15)				

### Overall survival analysis and association with Notch SNP and clinicopathological characteristics

**Fig. 5:**
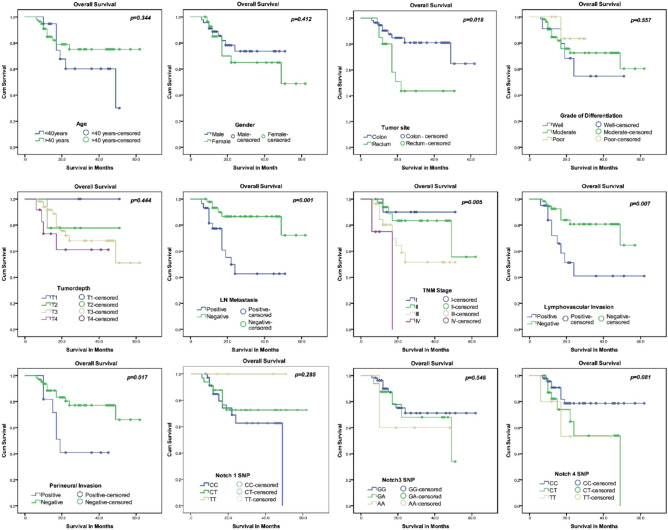
Kaplan–Meier analysis of overall survival in CRC patients shows a significantly worse survival in rectal tumor (*p* = 0.018), lymph node metastasis (*p* = 0.001), advanced stage (*p* = 0.005), presence of LNI and PNI (*p* = 0.007 and *p* = 0.017), and genotype with CT and TT in *Notch4* polymorphism (*p* = 0.081).

Of the 103 patients, 82 underwent resection. After histopathology, six patients did not have R0 resection and were excluded from the survival analysis (Table 4). We found that *Notch3* and *Notch4* polymorphism affected the prognosis of CRC patients. In *Notch3* gene polymorphism, homozygous mutant GG had lower 3-year survival (60%) compared to AA (71%) and AG (68%) types, although not significant (*p* = 0.546). Similarly, in *Notch4* polymorphism, patients with genotype CT (53.9%) and TT (53.3%) had a lower survival rate than CC (78.7%) (*p* = 0.081). Interestingly, Univariate analysis result showed that patient had significantly lower survival rate in rectal tumor (95% CI: 20.0–39.9, *p* = 0.018), presence of LN metastasis (95% CI: 22.7–38.5, *p* = 0.001), TNM stage III and IV (95% CI: 26.0–41.8 and 7.6–20.8, *p* = 0.005), presence of lymphovascular invasion (LVI) (95% CI: 21.9–46.5, *p* = 0.007) and perineural invasion (PNI) (95% CI: 17.8–34.6, *p* = 0.017). The Kaplan–Meier graphs representing the factors affecting overall survival are shown in Fig. 5.

**Table 4:** Kaplan–Meier analysis of CRC patients and association with *Notch* SNPs.

Parameters	n = 18 Event = Death	Mean Survival (%)	1-year survival(%)	3-year survival (%)	95%CI	*p-*value
Age						
< 40	07	36.9	94.7	60.2	28.7–45.2	0.344
> 40	11	49.9	84.8	75.2	43.5–56.3	
Gender						
Male	09	41.4	88.7	73.7	36.0–46.9	0.412
Female	09	43.4	85.0	65.0	34.0–52.8	
Tumor site						
Colon	09	50.81	90.3	81.0	44.2–57.4	0.018
Rectum	09	30.99	80.0	43.6	22.0–39.9	
Grade of differentiation						
Well	04	35.8	90.9	54.5	24.5–47.1	0.557
Moderate	13	47.0	84.5	72.4	40.1–53.9	
Poor	01	27.8	83.3	83.3	23.9–31.7	
Tumor depth						
T1	00	–	–	–	–	
T2	02	–	77.8	77.8	–	0.444
T3	12	–	91.8	68.0	–	
T4	04	–	73.3	61.1	–	
Lymph node						
Positive	12	30.6	77.4	42.6	22.7–38.5	0.001
Negative	06	53.6	93.2	86.6	47.4–59.7	
TNM Staging						
I	01	47.1	90.0	90.0	39.8–54.3	0.005
II	05	50.4	93.8	83.4	41.5–59.2	
III	10	33.9	80.2	51.5	26.0–41.8	
IV	02	14.2	75.0	–	07.6–20.8	
LVI						
Present	09	34.2	58.5	41.0	21.9–46.5	0.007
Absent	09	47.6	92.5	80.8	42.1–53.0	
PNI						
Present	06	27.1	81.8	40.9	17.8–36.4	
Absent	12	49.8	88.5	77.2	43.7–55.9	0.017
Notch1 SNP						
CC	10	–	84.7	62.6	–	
CT	08	–	87.8	72.5	–	0.285
TT	00	–	–	–	–	
Notch3 SNP						
AA	11	48.6	90.0	71.2	41.7–55.5	
GA	05	38.8	87.5	68.1	29.6–48.1	0.546
GG	02	32.8	60.0	60.0	16.4–49.1	
Notch4 SNP						
CC	08	51.8	90.8	78.7	45.5–58.1	0.081
CT	08	34.1	82.1	53.9	24.7–43.4	
TT	02	29.2	80.0	53.3	13.8–44.5

## Discussion

The present study looked at the association between Notch polymorphism, protein expression, and clinicopathological characteristics of CRC patients in the Indian population. The SNPs targeted in this study were located at various sites; rs3224591 is a 3’ UTR variant of *Notch1*, rs10910779 is a missense variant of *Notch2*, rs1043994 is a synonymous variant of *Notch3* and rs367398 is a 5’ UTR variant of *Notch4*. These genes of the *Notch* signalling pathway are involved in tissue repair, bone remodelling process as well as regulation of stem cells like self-renewal and epithelial-mesenchymal transition.

We found that the CT genotype of *Notch1* was associated with the risk of CRC compared to the CC genotype (OR 2.01 95% CI; 1.197–3.718). A similar finding was noted by Coa et al. in Breast cancer. They found that the frequency of rs3124591 CT genotype was significantly higher in breast cancer patients with invasive ductal carcinoma (IDC) and ductal carcinoma in-situ (DCI) compared to usual ductal hyperplasia controls (UDH)^23^. Overall, we did not find any significant difference in HWE between cases (HWE Pearson χ^2^ 0.085; HWE *p* = 0.988) and controls (HWE Pearson χ^2^ 20.91; HWE *p* = 0.000). Alanazi et al. also reported the polymorphism of *Notch1* rs3124591. However, they did not find any increased risk in the development of CRC as well as breast cancer in the Saudi population^22^. On clinical correlation, it was seen that the TT genotype in *Notch1* showed a significant association with increased depth of tumor (*p* = 0.035). Interestingly, the frequency of CT genotype was significantly associated with poorly differentiated tumors than well-differentiated and moderately-differentiated tumors in breast cancer^23^. This might point out that the T allele may be associated with differentiation and depth of invasion in cancer and can be explored further. Polymorphism rs10910779 is a *Notch2* missense variant resulting in a change in the amino acid sequence (Serine to Proline). However, we did not find polymorphism at this site in Indian CRC patients. The *Notch3* rs1043994 is a synonymous coding variant for alanine at codon 202. It regulates the transcription of the downstream target gene and plays an essential role in the development and maturation of most vertebrate organs and the survival of smooth muscle cells^24^. We found that the genotype distribution of *Notch3* showed deviation from HWE in cases suggesting an increased risk of developing CRC in the Indian population (*p* = 0.046). The frequency of AG and GG genotypes was associated with an increased risk of developing CRC than the AA genotype (*p* = 0.039). The frequency of the G allele also showed an association with the development of CRC (OR 1.697; 95% CI 1.001–2873; *p* = 0.049) compared to the A allele in our cohort. Similar findings were noted by Alanazi et al. in the Saudi population. They found that the genotype distribution of *Notch3* showed deviation from HWE^22^. Yagci et al. have reported an increased risk of having lung cancer with *Notch3* polymorphism in people of Turkish ancestry^25^. However, Cao et al. did not find any correlation between polymorphism in *Notch2* rs11249433 and *Notch3* rs1043994 with the risk of developing Breast cancer in the Chinese population^23^. Moreover, on clinical correlation, the *Notch3* polymorphism rs1043994 showed a significant association with the TNM stage (*p* = 0.013). *Notch4* polymorphism rs367398 is a non-coding transcript variant that plays an important role in vascular, renal, and hepatic development and can regulate stem cell-like self-renewal properties. The genotype frequencies of the heterozygous mutant (CT) and homozygous mutant (TT) were higher in patients than in the control group. However, there was no appreciable distinction between patients and controls in terms of the genotypes CT or TT. Clinical correlation showed an intriguing link between the frequency of the CT and TT genotypes and tumor differentiation with moderate and poor vs. well differentiation in our analysis (*p* = 0.039). We looked at *Notch4* rs367398 in its entirety, but no associations with clinical parameters were noted. This is the first study to assess the *Notch4* polymorphism in patients with CRC in Indian population. *Notch4* rs367398 polymorphism is not well characterized in CRC^26^. Anttila et al. reported patients with the *Notch4* CC genotype were ten times more likely to develop treatment resistance in schizophrenia patients. However, they did not find significant variations in *Notch4* frequencies between treatment-resistant and responsive patients^27^.

We also looked at the effect of *Notch* polymorphism at the translational level. We found that *Notch1* protein was markedly upregulated in tumor tissue compared to normal. The CT and TT genotypes showed increased protein expression in 45.23% and 57.14%, respectively. Similar changes were reported in previous studies^28,29^. Unlike *Notch1*, *Notch2* is essential for cell cycle regulation, hepatic cell development, and differentiation in morphologically healthy corpus epithelial tissue in humans and mice^30^. However, these cells may proliferate and dedifferentiate abnormally if Notch2 is overexpressed, which might eventually result in the formation of tumors and play a key role in oncogenesis. In this study, we detected increased expression of Notch2 in 42% of tumor tissue. However, this cannot be attributed to polymorphism. Notch3 is a well-known Notch receptor essential for controlling the proliferation, renewal, and rebuilding of colon epithelium as well as the conversion of stem cells into healthy mucosal epithelial cells. It has been shown that dysregulation of *Notch3* is associated with tumorigenesis in various cancers. In breast cancer, *Notch*3 primarily functions as an oncogene, with a few exceptions. Fernandez et al. reported increased expression of *Notch3* in the human inflammatory Breast cancer xenograft model^31^. A study on 74 squamous cell carcinoma (SCC) patients of the oral cavity showed increased Notch3 expression in tumors compared to the normal epithelial tissues^32^. Previous data suggest that the expression of *Notch3* is higher in tumor tissue than normal tissue in various solid tumors, including hepatocellular carcinoma, CRC, and gallbladder cancer^33–35^. Our Notch3 western blot result showed significantly increased expression in 73.07% of the mutant genotype AG and 75% in GG (*p* = 0.047). Furthermore, we found a significant association (*p* = 0.047) with *Notch3* polymorphism A > G and elevated protein expression in tumor tissue and increased risk of CRC development. These findings suggest that *Notch3* might be a therapeutic target for CRC with AG and GG genotypes. The GG type was also associated with poor survival, although it was not statistically significant. Further studies are required to prove the importance of *Notch3* as a prognostic biomarker. The increased protein expression in *Notch4* with CT (43.24%) genotype and mutant TT( 50%) are in line with previous studies by Wu et al. in the Chinese population^36^. Xiu et al. concluded that increased *Notch4* expression was associated with tumor development^37^. *Notch4* regulates a number of tumor cell behaviors, such as epithelial-mesenchymal transition (EMT), radio or chemoresistance, angiogenesis, and stem cell-like self-renewal (CSCs)^36,38^. Furthermore, *Notch4* is crucial for the development of colorectal CSCs as it regulates the transcription factors that are linked to stemness^39^.

The survival analysis revealed that rectal tumors (*p* = 0.018), lymph node positivity (*p* = 0.001), advanced stage (*p* = 0.005), lymphovascular invasion (*p* = 0.007), and perineural invasion (*p* = 0.017) are predictors for poor survival outcomes in CRC. Looking at the prognostic implications of *Notch* genes in CRC, we noted that patients with GG genotype in *Notch3* and TT/ CT type in *Notch4* had poor survival. Although not significant, it could be considered a prognostic marker for poor survival outcomes in CRC after further validation in a large cohort. Moreover, it is yet unknown what biological processes underlie the associations between CRC and alterations in the *Notch* gene family; an in-vitro assessment of the CRC cell line and further studies using high-throughput sequencing and epigenetic profiling would help unravel the therapeutic benefits of targeting Notch receptors for managing CRC patients.

## Conclusions

Among Notch receptors, *Notch3* showed an association with an increased risk of developing CRC in the Indian population, and *Notch3* SNP has shown a significant association with increased protein expression. On clinical correlation, *Notch3* (rs1043994) A > G is associated with advanced disease stage in CRC, while *Notch1* and *Notch4* showed an association with depth of invasion and tumor grade, respectively. *Notch3* and *Notch4* variants showed worse survival. This finding suggests that *Notch3* and *Notch4* polymorphism could be a potential indicator for the development of CRC with some prognostic value. However, further study on a larger sample size will be required to validate our findings.

## Supplementary Information

Supplementary Information.

## Data Availability

Sanger sequencing data that we analyzed to support our findings have been submitted to GenBank under submission ID #2,862,335. The datasets used and/or analyzed during the current study are available from the corresponding author upon reasonable request. The whole-exome sequencing data is available in the Sequence Read Archive (SRA) under BioProject accession PRJNA1079014. (https://www.ncbi.nlm.nih.gov/bioproject/PRJNA1079014).
