# Supplementary material for: Role of Notch gene receptors as prognostic biomarkers in colorectal cancer
Source: Sci Rep. 2025 Sep 30;15:33782. doi: 10.1038/s41598-025-00424-5 (PMC12485164; doi:10.1038/s41598-025-00424-5)
Supplement: Supplementary file 1 — Supplementary Information. [file 41598_2025_424_MOESM1_ESM.pdf]

Supplementary File

Title: "Role of Notch gene receptors as prognostic biomarker in Colorectal cancer"

Journal: International Journal of Colorectal Disease

Authors: Abhay Kumar Sharma[1,2], Nimisha Nimisha[2], Arun Kumar[3], Apurva Apurva[2], Abhishek Kumar[1], Ejaj Ahmad[2], Asgar Ali[4], Birendra Prasad[1], Sundeep Singh Saluja[2,5*]

[1] Department of Botany/Biotechnology, Patna University Patna, India.

[2] Central Molecular Lab, Govind Ballabh Pant Institute of Postgraduate Medical Education and Research (GIPMER), New Delhi, India.

[3] Department of Biotechnology, Amity University Noida, India

[4] Department of Biochemistry, All India Institute of Medical Sciences Patna, India

[5] Department of G I Surgery, Govind Ballabh Pant Institute of Postgraduate Medical Education and Research (GIPMER), New Delhi, India.

Corresponding Author

Dr. Sundeep Singh Saluja

PI Central Molecular Lab,

Director Professor, Department of GI surgery,

Govind Ballabh Pant Institute of Postgraduate Medical Education and Research (GIPMER)

1, Jawaharlal Nehru Marg, 64 Khamba, Raj Ghat, New Delhi, India

Phone: +91-9718599259

Email: sundeepsaluja@yahoo.co.in

Supplementary table 1: Details of Restriction digestion of *Notch* and their digested products.

Gene	Restriction Enzyme	Restriction digestion condition	Fragment pattern in (bp)		
Notch1	MnII	37°C for 15 min, inactivation 65°C for 20 minutes	CC (397+43)	CT (440+397+43)	TT (440)
Notch2	MnII	37°C for 15 min, inactivation 65°C for 20 minutes	TT (228+100)	TC (190+100+38)	CC (190+38)
Notch3	MwoI	37°C for 15 minutes	AA (168+61)	AG (168+107+61)	GG (107+61)
Notch4	MspI	37°C for 15 minutes	CC (190+40)	CT (230+190+40)	TT (230)

Supplementary table 2: Clinico-pathological characteristics of CRC patients

Clinicopathological characteristics	Group	Case (n = 103)	Control (n=103)
Age	≤ 40	27	30
	> 40	76	73
Gender	Male	59	55
	Female	44	48
Site	Colon	66	-
	Rectum	37	-
Grade of Differentiation	Well	12	-
	Moderate	77	-
	Poor	14	-
TNM Stage	I	10	-
	II	44	-
	III	42	-
	IV	07	-
Tumor Depth	T1	03	-
	T2	09	-
	T3	67	-
	T4	24	-
Lymph Node Metastasis	Positive	41	-
	Negative	62	-
Lymphovascular Invasion	Positive	23	-
	Negative	80	-
Perineural Invasion	Positive	13	-
	Negative	90	-

Supplementary table 3: Hardy-Weinberg equilibrium deviation test for Notch1-4

Genotype	Case n (Freq.)	HWE Pearson $\chi2$	HWE p-value	Control n (Freq.)	HWE Pearson $\chi2$	HWE p- value
Notch1 (rs3124591)						
CC	54 (52.42)	0.085	0.988	39 (37.86)	20.91	0.000
CT	42 (40.77)			64 (62.14)		
TT	07 (06.79)			00 (00.00)		
Notch2 (rs10910779)						
TT	103 (100)	NA	NA	103 (100)	NA	NA
Notch3 (rs1043994)						
AA	69 (67.99)	6.128	**0.046**	80 (77.66)	3.900	0.142
AG	26 (25.24)			19 (18.44)		
GG	08 (07.76)			04 (03.88)		
Notch4 (rs367398)						
CC	58 (56.31)	0.370	0.831	64 (62.13)	0.416	0.0812
CT	37 (35.92)			33 (32.03)		
TT	08 (07.76)			06 (05.82)		

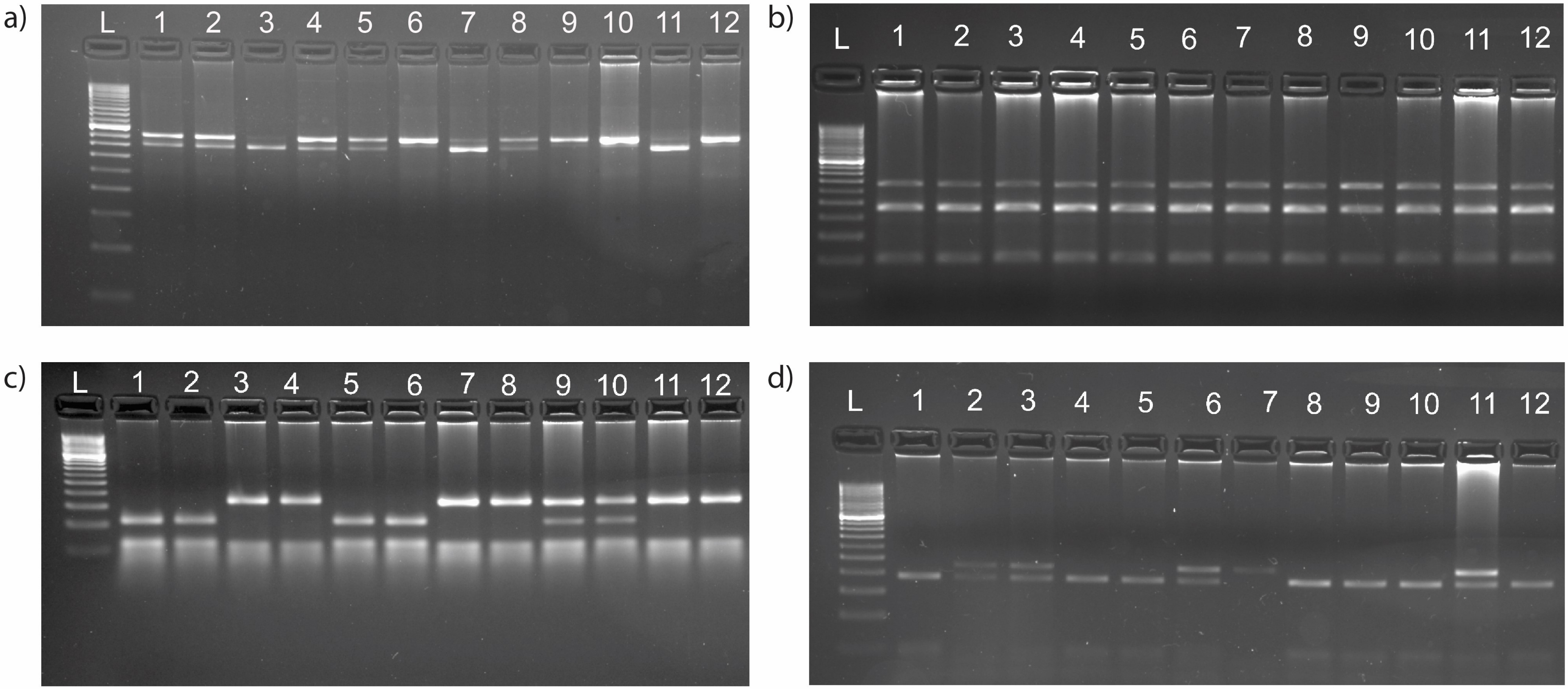
a)
L 1 2 3 4 5 6 7 8 9 10 11 12
b)
L 1 2 3 4 5 6 7 8 9 10 11 12
c)
L 1 2 3 4 5 6 7 8 9 10 11 12
d)
L 1 2 3 4 5 6 7 8 9 10 11 12

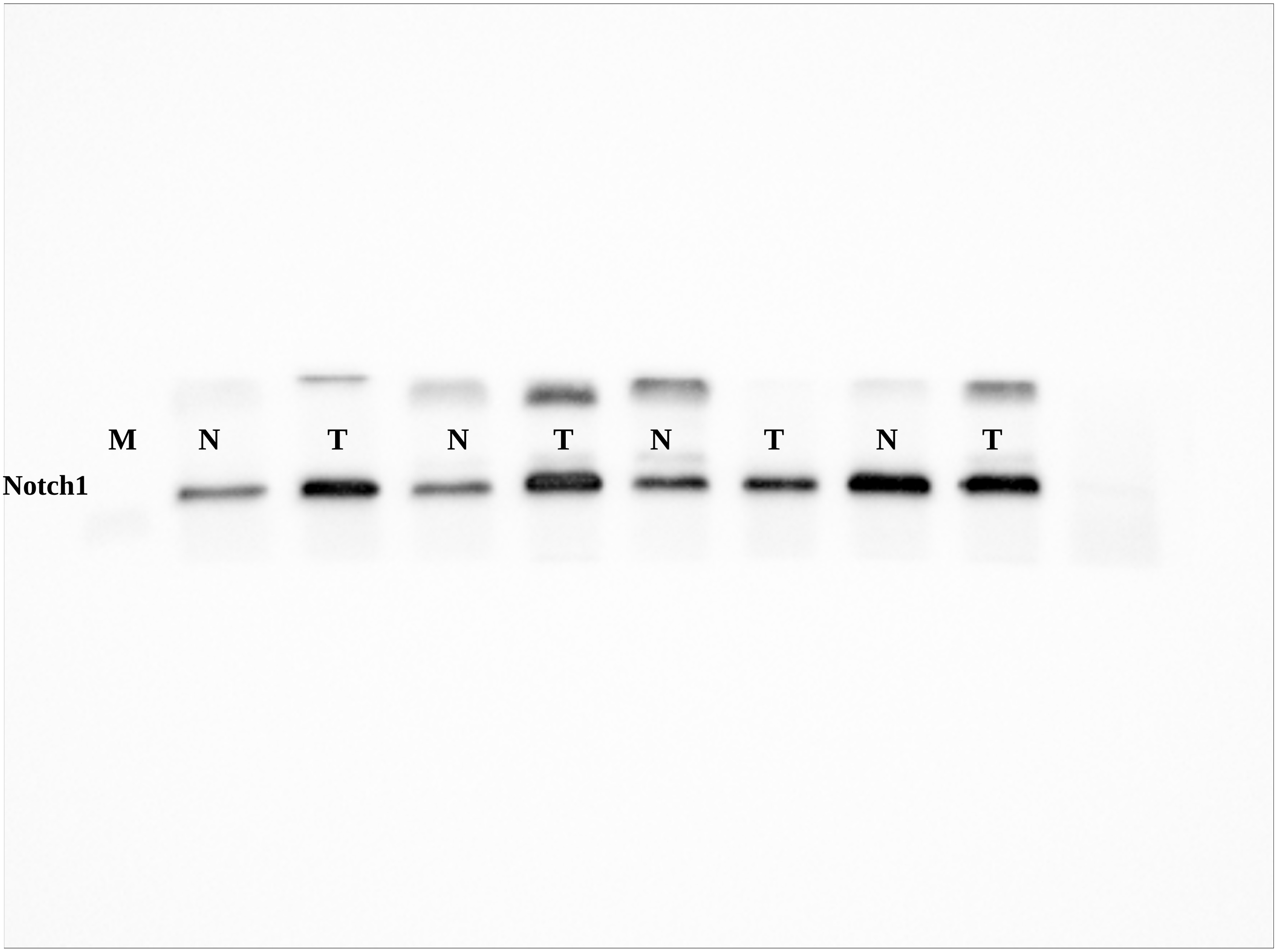
M N T N T N T N T
Notch1

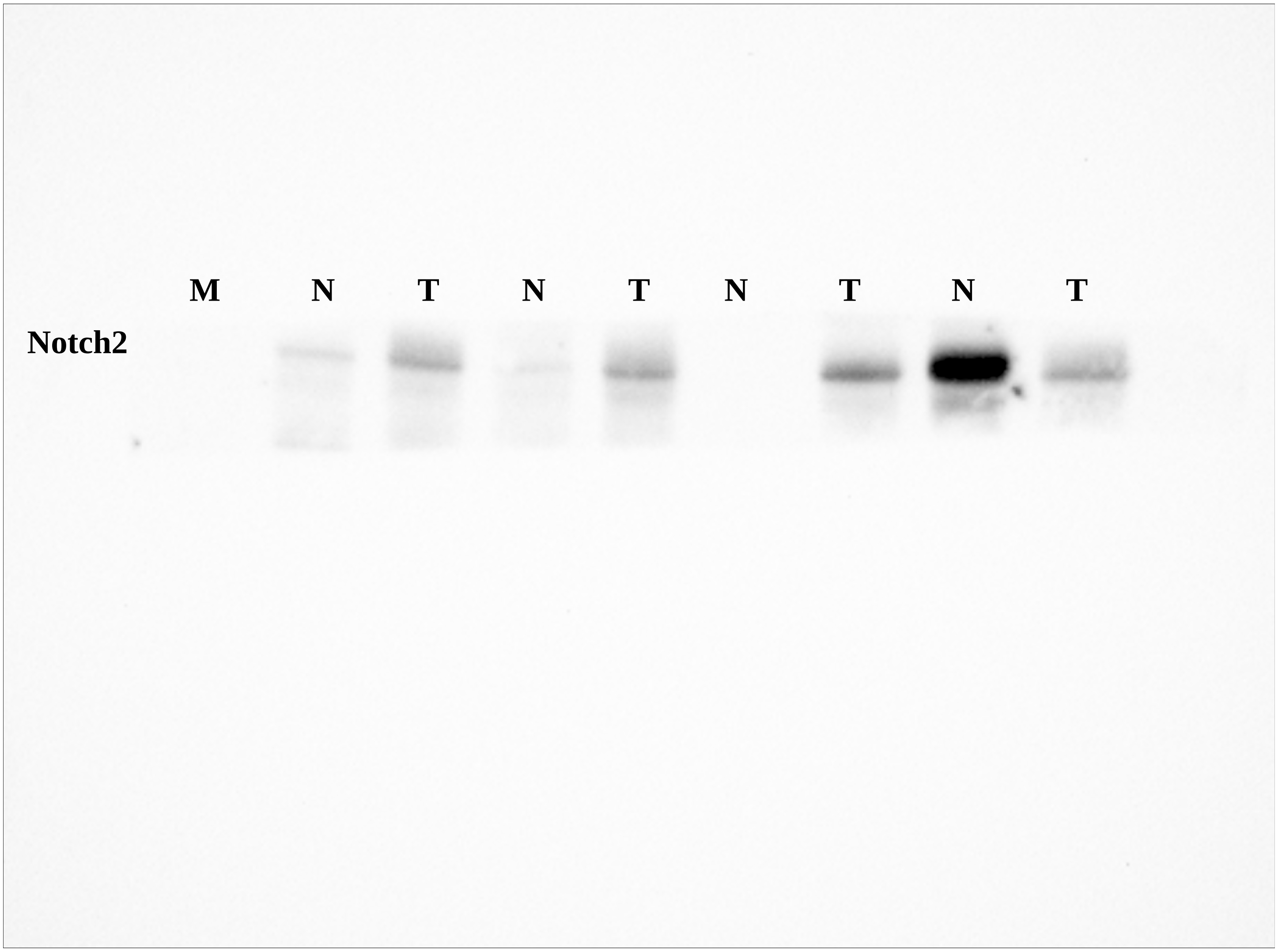

M N T N T N T N T
Notch2

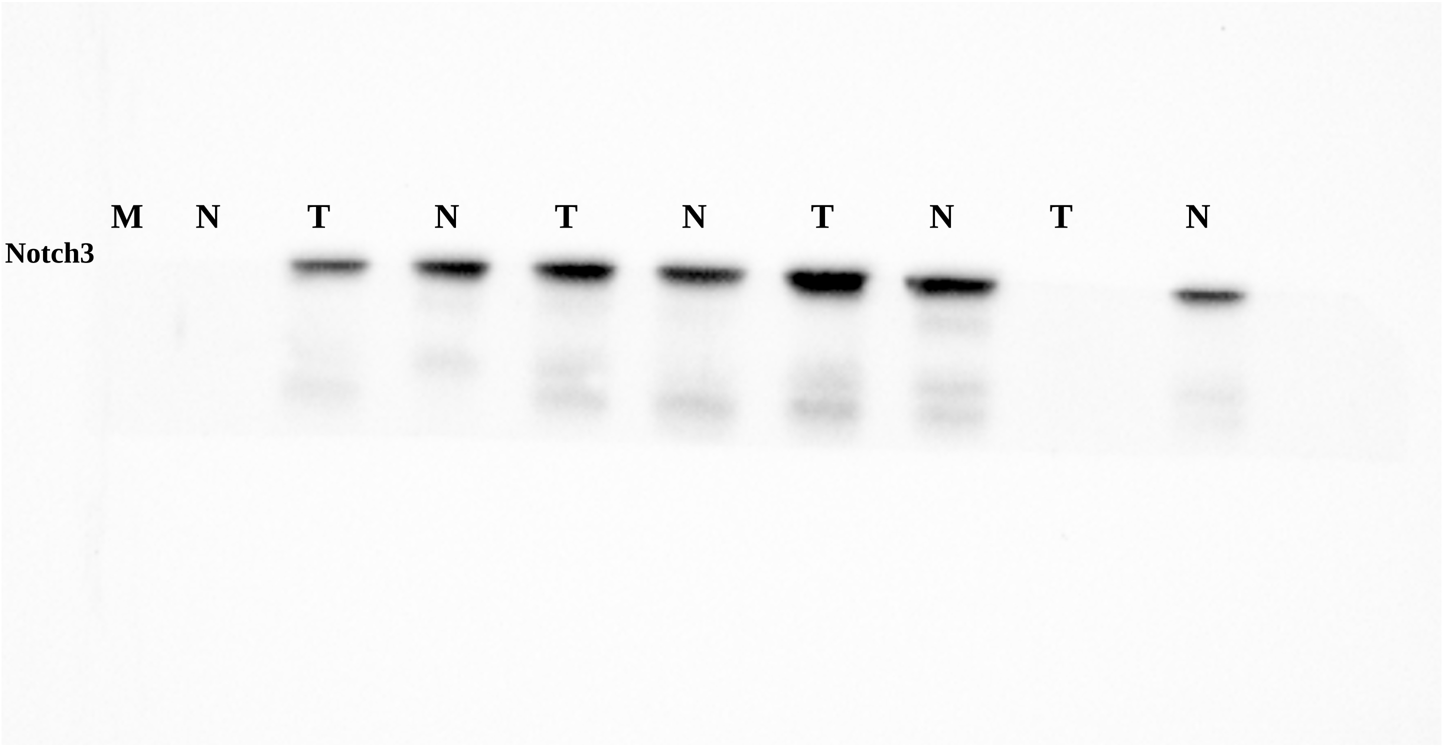
M N T N T N T N T N
Notch3

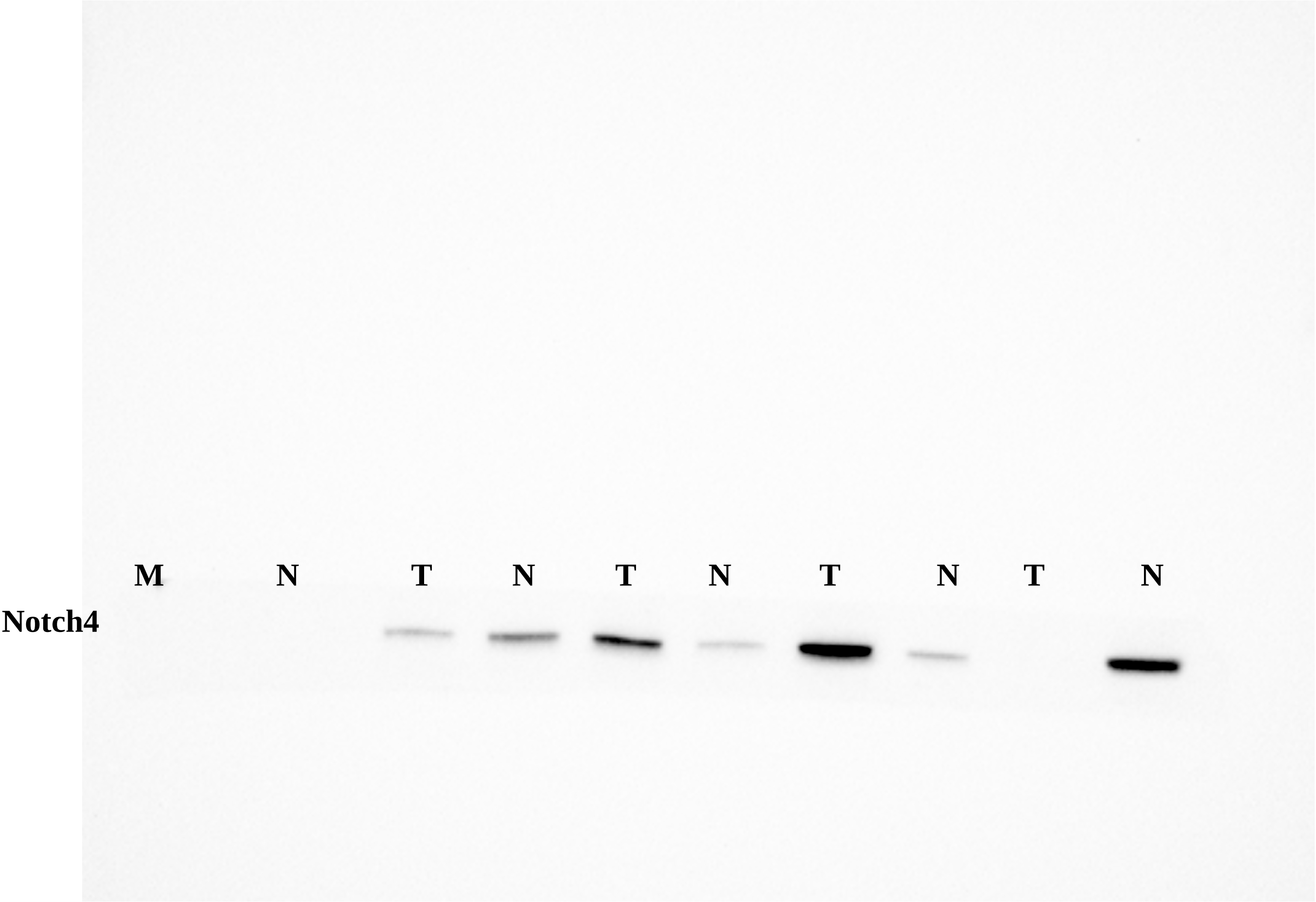
M
N
T
N
T
N
T
N
T
N
Notch4

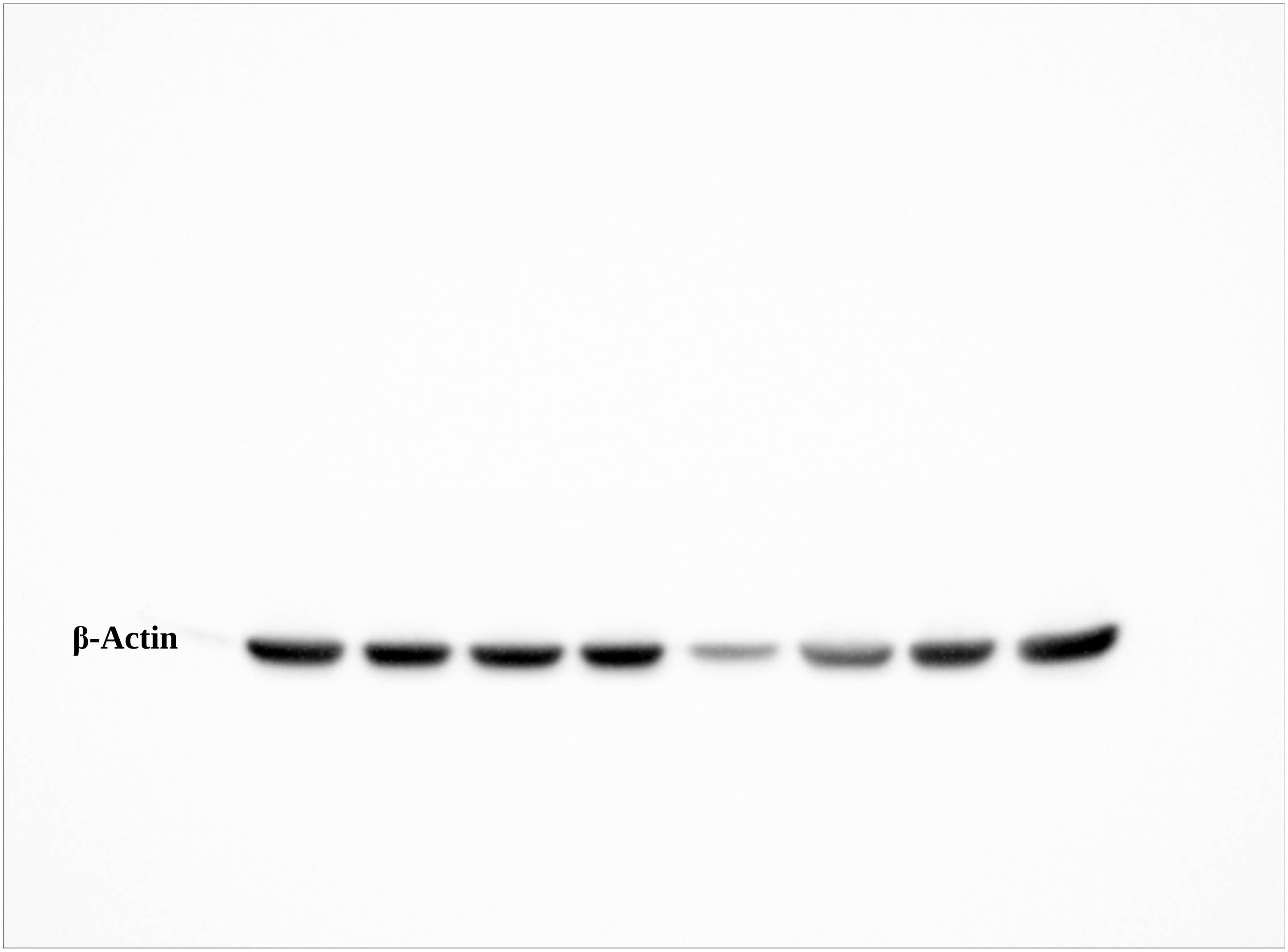
β-Actin

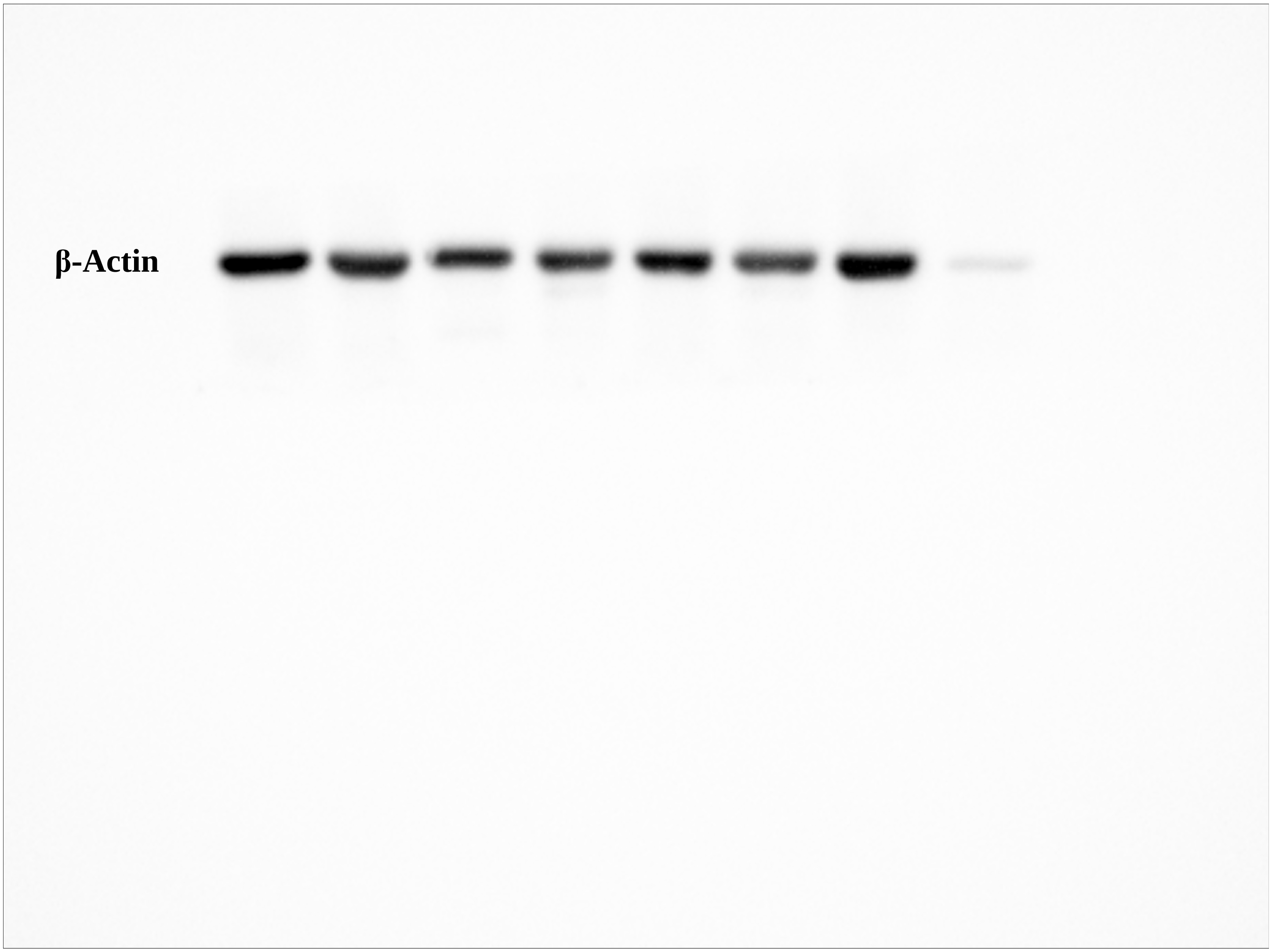
β-Actin
